# HistoEncoder: A digital pathology foundation model for prostate cancer

**DOI:** 10.1016/j.jpi.2026.100715

**Published:** 2026-08-31

**Authors:** Joona Pohjonen, Oussama Batouche, Janne Kantola, Antti Rannikko, Kevin Sandeman, Andrew Erickson, Esa Pitkänen, Tuomas Mirtti

**Affiliations:** a Research Program in Systemic Oncology (ONCOSYS), Faculty of Medicine, University of Helsinki, Helsinki, Finland; b Doctoral Program in Computer Science, Faculty of Science, University of Helsinki, Helsinki, Finland; c Institute for Molecular Medicine Finland (FIMM), HiLIFE, University of Helsinki, Helsinki, Finland; d Research Program in Applied Tumour Genomics, Faculty of Medicine, University of Helsinki, Helsinki, Finland; e iCAN Digital Precision Cancer Medicine Flagship, Helsinki, Finland; f Department of Urology, Helsinki University Hospital, Helsinki, Finland; g Department of Pathology, Division of Laboratory Medicine, Skåne University Hospital, Malmö, Sweden; h Department of Pathology, Helsinki University Hospital, Helsinki, Finland; i Finnish Cancer Institute, Helsinki, Finland

**Keywords:** Foundation models, Prostate cancer, HistoEncoder, Digital pathology, Prognosis prediction, Self-supervised learning

## Abstract

Foundation models are trained on massive amounts of data to capture complex patterns in images. Subsequently, a wide range of downstream tasks can be adopted with minimal computational resources. We have developed HistoEncoder, a foundation model for prostate cancer digital pathology by pre-training on 48 million prostate tissue tile images. HistoEncoder allows automated extraction of histological features highly predictive of Gleason patterns achieving comparable performance with substantially larger pan-cancer foundation models while being much more efficient. By fine-tuning the model with a small amount of data and computational resources, we describe two clinical use cases for HistoEncoder. First, HistoEncoder can be used to automatically annotate large-scale datasets with high accuracy. Second, we show that HistoEncoder-derived histology clusters contain prognostic information of a similar magnitude to Gleason grading in internal cross-validation. Lightweight foundation models such as HistoEncoder allow organizations to build effective clinical software tools without the need for extensive datasets and heavy computing.

## Highlights

• HistoEncoder is a foundation model trained and validated with prostate tissue images.
• Domain-specific model outperforms natural-image models with minimal fine-tuning.
• The open-source model enables large-scale tissue annotation for downstream tasks.
• HistoEncoder features contain prognostic information of a similar magnitude to Gleason grading.

## Introduction

Digital histopathology (DP) has been at the forefront of adopting artificial intelligence (AI) methods due to existing well-developed practices to digitize and process abundantly available sample slides in clinical laboratories. In DP, convolutional neural networks^1^, ^2^ (CNNs) trained in a supervised fashion have reached non-inferiority compared with human observers in clinical tasks such as cancer detection, grading, and somatic mutation detection.^3^, ^4^, ^5^ Many of the published classification models in DP have been trained by transfer learning,^6^ utilizing networks pre-trained with natural images, which are then fine-tuned for downstream tasks. Still, due to the considerable domain differences between histological and natural images, transfer learning from a neural network pre-trained with natural images (such as ImageNet^7^) offers little benefit to performance. Another challenge with the supervised approach has been the need for large datasets with expert annotations. This has led to growing interest in unsupervised and self-supervised learning (SSL) approaches as alternatives.^8^, ^9^, ^10^ Yet another challenge is the generalizability of current AI models across the variation caused by different scanners and lab circumstances. The effect of this kind of domain shift is best controlled by domain-specific methods.^11^, ^12^ Ideally, DP models for novel prognostic or predictive feature extraction should be trained with techniques that do not require meticulous expert annotations and manage to overcome the challenges in domain shift.

Very large neural network models, also called foundation models (FMs)^13^, ^14^ have shown exceptional performance in protein structure and variant effect prediction,^15^, ^16^ natural language modeling,^4^ and image analyses.^17^, ^18^, ^19^ These models are trained with SSL on large data and often fine-tuned to a specific task with domain-specific data.^13^ FMs leverage the abundance of unlabeled data via SSL,^18^, ^19^, ^20^, ^21^ and can be then easily adapted for downstream tasks, even without any additional training.^17^, ^22^, ^23^ FMs pre-trained on medical data may facilitate the development of clinically useful methods for precision cancer medicine.

Prostate cancer (PCa) is the second most commonly diagnosed cancer in men worldwide,^24^ but many localized tumors follow an indolent clinical course and will not threaten the patient's life. Treatment decisions must therefore balance tumor aggressiveness and the probability of PCa-specific progression or death against the patient's life expectancy, health status, comorbidity, and risk of death from competing causes.^25^ In localized disease, a life expectancy of more than approximately 10 years is generally required to derive meaningful benefit from curative treatment, whereas active surveillance or watchful waiting can avoid unnecessary treatment-related morbidity in appropriately selected patients. ISUP Grade Group, serum prostate-specific antigen, and clinical stage remain central to current risk stratification.^25^ Histopathology-based Gleason grading has suffered from grade migration and inflation, leading to clustering of new diagnoses into GG2 and GG3.^26^ Substantial outcome heterogeneity remains within intermediate-risk disease, warranting for better risk stratification for more individualized treatment decisions.

Deep learning systems have achieved pathologist-level performance in PCa detection and Gleason grading in both radical prostatectomy specimens and diagnostic needle biopsies, including in independent and external validation cohorts.^27^, ^28^, ^29^, ^30^ Large-scale international evaluation in the PANDA challenge further demonstrated that several independently developed algorithms could generalize across patient populations, labs and reference standards.^31^ AI assistance has also improved the agreement of pathologists with expert reference grading.^32^ However, most clinically oriented systems have focused on cancer detection and reproduction of Gleason grading. A potentially greater added value of computational pathology is the identification of morphological features beyond conventional glandular architecture that may provide more granular prognostic information and guide treatment selection, particularly for patients with intermediate-risk disease.

Variability in the PCa tissue architecture vastly exceeds the categorical GG determined by pathologists or AI-supported models, whether based on diagnostic biopsies or surgical specimens.^33^ It is expected, and the object of this study, that AI-based methods have the potential to highlight these morphologies and classify cancer with improved prognostic granularity. Additionally, AI offers new opportunities to combine multiple modalities for risk-classification and prognostic work-up.

Biochemical recurrence (BCR) is considered a cancer relapse after primary treatment and often leads to secondary therapies. The incidence of BCR is as high as 30%–50% but only 10%–15% of these men ultimately die of PCa. Some earlier studies have addressed different DL models in the risk-assessment for BCR, which is, however, considered as a surrogate end point to more clinically significant end points such as metastasis-free survival or cancer-specific death. In an earlier study, Wulczyn et al. showed that AI-derived quantitative Gleason grading can predict PCa-specific mortality in a large radical-prostatectomy cohort with long-term follow-up.^27^ These studies demonstrate the prognostic potential of tissue-image analysis, although they are task-specific supervised models rather than reusable FMs. To our knowledge, no prostate-specific FM has previously been adapted to prediction of PCa-specific mortality.

In this study, we construct HistoEncoder (Fig. 1), a FM for DP pre-trained with 48 million prostate tissue tile images from thousands of histological whole-slide images (WSI). We show how HistoEncoder can better distinguish malignancy in prostate tissue images than FMs pre-trained with natural images even without fine-tuning. HistoEncoder performs at a level comparable to pan-cancer FMs, which are an order of magnitude larger and thus, require substantially more resources than HistoEncoder while being much slower to use. We then demonstrate two applications of HistoEncoder in PCa medicine. First, we utilize HistoEncoder to annotate one of the largest publicly available histological dataset on PCa with high accuracy.^28^ Second, we evaluate HistoEncoder-derived histology cluster fractions as predictors of PCa-specific mortality and compare their discrimination directly with conventional Gleason grading. Lastly, we discuss how HistoEncoder can be extended to address other challenges of precision cancer medicine with minimal effort and data.

**Fig. 1:**
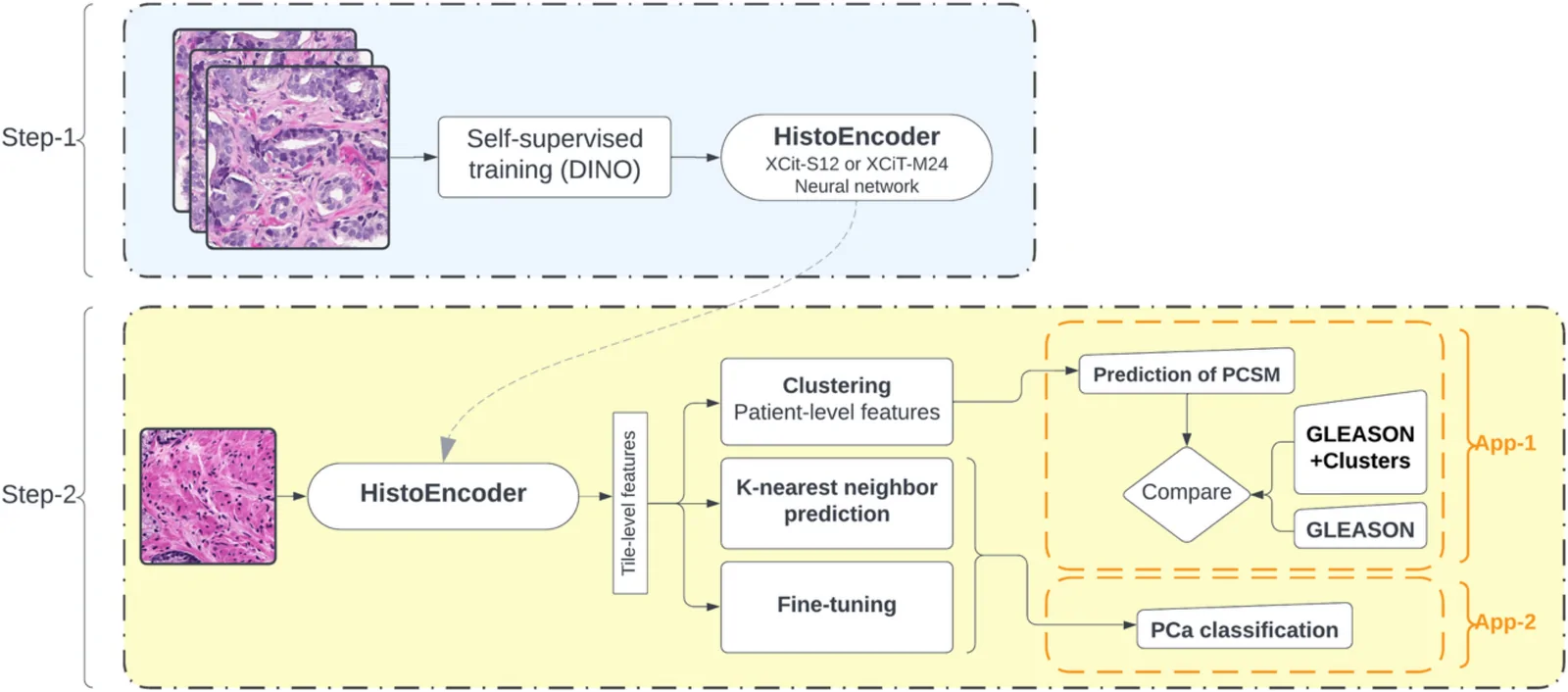
An overview of the HistoEncoder workflow. HistoEncoder utilizes the cross-covariance transformer (XCiT) as the backbone network. The models are pre-trained with 48 million prostate tissue tile images from 1307 patients in a self-supervised manner (Step 1), and fine-tuned to cancer classification and mortality prediction tasks, or used without fine-tuning via a KNN classifier (Step 2).

## Methods

### Training HistoEncoder models

In this study, we train cross-covariance image transformers (XCiT)^28^ with a SSL method DINO,^22^, ^29^ which leverages discriminative signals between groups of images to learn useful features using self-distillation. Instead of focusing on maximizing the fine-tuning performance on a downstream task,^30^, ^34^ the encoder model training aims to maximize the representativeness of extracted features.^22^ We selected XCiT due to linear complexity in the number of tokens, allowing efficient processing of high-resolution images,^28^ which are commonly encountered in digital pathology. Two XCiT backbones are trained on prostate tissue samples, prostate-s and prostate-m, based on the small- and medium-sized XCiT model variants XCiT-S12 and XCiT-M24, respectively.

Many models for clinical digital pathology image analysis have been pre-trained with natural images, often using supervised training methods.^35^, ^36^, ^37^, ^38^, ^39^, ^40^, ^41^, ^42^, ^43^, ^44^, ^45^, ^46^ Although it is common to use models pre-trained with supervised training methods, this does not always produce useful embedded features, when compared to models trained with self-supervised methods (see Caron et al.,^29^ and Suppl. Tables 1, 2, and 3). Thus, to make comparisons between models trained with histological images and natural images fairer, all comparisons are against XCiT-S12 and XCiT-M24 models trained on natural images using the self-supervised method DINO.^7^, ^29^ These models are denoted as natural-s and natural-m, respectively.

### Workflows leveraging encoder models

We describe two workflows that leverage the image features from our encoder models. The first is for automatic pre-annotation of large imaging datasets, and the second is for combining encoder-derived histological features with conventional Gleason grading. Command line interfaces and a Python module for running these workflows can be found in the GitHub repository https://github.com/jopo666/HistoEncoder.

#### Automatic annotation of large-scale slide image datasets

First, all slide images in the dataset are cut into small tile images with HistoPrep.^47^ Second, features for all tile images are extracted with either prostate-s or prostate-m encoder models. Third, all extracted features for the tile images are clustered into *n* clusters with mini-batch K-means. Given that HistoEncoder produces similar embedded features for tile images with similar histological patterns, clusters can be labeled based on visual inspection of the prevailing histological pattern.

After automatically pre-annotating all tile images in a dataset with HistoEncoder, a pathologist can visually inspect a subset of the tile images in each cluster. Now, if a given cluster contains only a single histological pattern, such as stroma or epithelium, all tile images assigned to this cluster can be labeled based on the prevailing pattern. If a cluster contains too much variability, the tile images in this cluster can be further clustered and labeled to produce clusters with less variability. The process can then be repeated until enough tile images have been labeled for the task at hand.

#### Combining histomics with other data modalities

Here, we present a workflow to integrate HistoEncoder image features with other data modalities. When additional data modalities, like spatial transcriptomics data^48^ or tissue microarray (TMA) spot labels, provide information for smaller tissue regions, feature vectors can be directly generated for these regions using the prostate-s or prostate-m encoder models.

In contrast, if the data modalities to be integrated contain information for larger tissue regions or WSIs or multiple slide images, the slide images must be cut into smaller tile images that are compatible with HistoEncoder. A common example is patient-level clinical data, where data from each patient pertains to all tile images from the same patient.

In this case, we will first automatically annotate and cluster all tiles. Each cluster contains tile images with similar histological patterns. Second, we will calculate, for each patient, the proportion of tile images assigned to each cluster (“cluster fractions”). Cluster fractions represent a patient-specific summary of histological patterns observed in the slide images.

To give an example, in a dataset of tissue biopsy slide images and associated clinical data, we can derive patient-level cluster fractions by analyzing all tile images extracted from each biopsy slide. If a specific cluster predominantly contains tile images with high-grade cancer, the fraction of this cluster for a given patient would represent the proportion of high-grade cancer observed in that patient's biopsy slides.

#### Comparative evaluation of tile-level representations against other models

To evaluate the quality of tile-level representations learned by HistoEncoder, we compared features extracted by prostate-s and prostate-m against six other models on the Radboud dataset. Four recently released pathology FMs, H-Optimus-1,^49^ UNI2-h,^49^ Virchow2,^49^ and CONCH v1.5^50^ were chosen for this comparison, as well as natural-s and natural-m.

The images in the Radboud dataset were tiled into 224-pixel patches, extracted at 20× magnification before feature extraction with Trident.^51^ Each tile was then labeled as either benign, Gleason 3, Gleason 4, and Gleason 5 based on the annotations included in the Radboud dataset. The label for each tile was decided by the dominant annotation and additionally for linear probing, only tiles where the dominant annotation comprised at least 50% of the tile were retained for analysis.

Extracted features were visualized with two-dimensional UMAP^52^ plots in order to qualitatively compare the feature spaces created by each model. For computing the UMAP embeddings, a maximum of 90 tiles were sampled from each slide, with tiles from each class being sampled equally. In total 454,379 tiles were sampled from 5060 slides. The UMAP embeddings were computed using RAPIDS cuML,^53^ with *n_neighbors = 15 and min_dist* = 0.1.

The quality of the feature vectors extracted by each model was quantitively estimated by training logistic regression classifiers on the feature vectors extracted by each model (i.e.*,* linear probing) to assign image tiles into one of four tissue classes (benign, Gleason 3/4/5)*.* The data were split 80/20 at the slide-level, stratified by the four classes. Features were normalized to have zero mean and unit variance by fitting the scaling parameters on the training set. The L-BFGS solver^52^ was used together with L2 regularization for each linear probe. Additionally, the inverse L2 regularization strength C was set to *dk*/100, where *d* is the embedding dimension for each model and *k* is the number of classes, following the example by Chen et al.^54^ After training each probe, per-class precision, recall, F1, and area under the receiver operating curve (AUROC) were calculated on the test set. Confidence intervals for the metrics were calculated with bootstrap resampling of the test set predictions over 10,000 iterations.

In order to evaluate the computational cost of HistoEncoder, the per-slide feature extraction times of prostate-m were compared against H-Optimus-1, UNI2-h, Virchow2, and CONCH v1.5. For the data in this comparison, slides from the HelsinkiTMA dataset were tiled into 512px patches extracted at 80× magnification using Trident. In total, there were 713,239 tiles from 1769 images.

Feature extraction was run for each model with a single NVIDIA Tesla P100 GPU. Because the images were processed sequentially, the per-slide feature extraction times were estimated from the creation timestamps from two subsequent output files for each image. The first images for each model were excluded from the analysis. For each model, the relative difference in per-slide processing time was calculated by dividing its mean per-slide time by that of prostate-m. Statistical significance was calculated using paired *t*-tests between prostate-m and the other models.

### Datasets

All datasets leveraged in this study are summarized in Table 1. All slide images are processed with HistoPrep.^47^

**Table 1:** Datasets used in this study.

Name	Medical center	Patients	Slides	Type	Tile images^a^	Ref
HelsinkiProstate	Helsinki University Hospital	1307	11,226	Biopsy & Organ section	898.4 million	–
Helsinki30^b^	Helsinki University Hospital	30	465	Organ section	75.2 million	^55^
Helsinki60^b^	Helsinki University Hospital	60	863	Organ section	146.0 million	^55^
HelsinkiTMA	Helsinki University Hospital	432	1769	Tissue microarray	0.5 million	–
PESO	Radboud University Medical Center	40	137	Region of interest	13,287	56, 57
Karolinska	Karolinska Institutet	2113	5456	Biopsy	3.2 million	^31^
Radboud	Radboud University Medical Center		5160	Biopsy	2.6 million	^31^

a Non-overlapping 256 × 256 tiles with >50% tissue.

b Part of HelsinkiProstate dataset.

#### Training dataset

Training data, designated as HelsinkiProstate, for the prostate tissue encoders, consists of patients who have undergone prostate biopsies or radical prostatectomy in Helsinki University Hospital between 2013 and 2021. In total, there are 1307 patients with 5642 and 5584 tissue slides of needle biopsies, and tissue sections from radical prostatectomy (Table 1). To create the HelsinkiProstate dataset, each slide is cut into 640 × 640 pixel tile images at magnifications 20×, 10×, and 5× with 20% overlap between neighboring tile images. This produces several hundred million tile images, which are then preprocessed with HistoPrep^47^ to remove tiles containing non-tissue areas such as pen markings or other artefacts. Once irrelevant tile images have been filtered out, all remaining tile images are run through a PCa classifier model from Pohjonen et al.^55^ To create a balanced training dataset, all 16 million tile images with a prediction score ŷ > 0.2, and 32 million randomly sampled tile images with score ŷ ≤0.2 were selected to comprise the HelsinkiProstate dataset.

#### Evaluation datasets

HelsinkiTMA dataset contains 1769 TMA spots from 432 PCa patients who underwent radical prostatectomy between 1983 and 1998 at the Helsinki University Hospital. All slide images are cut into 512 × 512 with 25% overlap between neighboring tiles. The patients have a median follow-up time of 19.0 years. Survival analysis included 398 patients with complete disease-specific survival follow-up, Gleason grade, and linked HistoEncoder cluster-fraction data. Of these patients, 52 died from PCa.

Helsinki30 and Helsinki60 datasets^55^ contain WSIs (normal size and whole mounts) from 30 and 60 patients who have undergone radical prostatectomy at the Helsinki University Hospital between the years 2014 and 2021. All slide images in both datasets have been annotated by pathologists and classified as cancerous or benign. A portion of these datasets were also part of the training (HelsinkiProstate) dataset (Table 1).

Several publicly available datasets are used in this study. The test set of the PESO dataset^56^, ^57^ contains 137 tile images of 2500 × 2,500 pixels from 37 different slide images annotated as either cancerous or benign. PANDA development dataset^31^ contains 10,616 prostate biopsy slides from 2113 patients from Radboud University Medical Center and Karolinska Institute, which we denote in this study as Radboud and Karolinska.

### Fine-tuning the encoder models

To fine-tune the encoders for downstream tasks, the partial fine-tuning setup from He et al.^30^ is used, with the following modifications. RandAugment^58^ is replaced with StrongAugment^55^ 2, 3, or 4 operations with 0.5, 0.3, and 0.2 probability, respectively. Each image is flipped vertically and/or horizontally, and a perspective operation is applied with a scale from 0 to 0.1 with a probability of 0.5. For partial fine-tuning,^30^ only the last 0.5, 2, 4, or 8 XCiT-S12 model blocks are fine-tuned, where 0.5 denotes fine-tuning the last fully connected layer of the last model block. There are 1.182, 3.550, 7.120, and 14.260 million fine-tuned parameters for 0.5, 2, 4, and 8 model blocks, and 25.868 million for the full XCiT-S12 model. For fine-tuning, the encoders without training, a K-nearest neighbor (KNN) classifier is used with k = 20. The KNN-classifier is fit with the features extracted from the training dataset, and label predictions correspond to the proportion of nearest neighbors with a positive label. Each experiment is repeated five times, and the mean and standard deviation of the AUROC are reported.

For the PESO dataset, randomly resized crops with scale [0.01, 0.2] are randomly sampled from the 2500 × 2500 pixel region of interest images during fine-tuning, and an epoch is defined as 51,200 training samples. For Karolinska and Radboud datasets, training images are randomly sampled from 384 × 384 pixel tile images with 20% overlaps, and an epoch is defined as 262,144 training samples, or the maximum amount of tile images included in model training. When limiting the number of training images from the PESO dataset, region of interest images from 1, 2, 4, 8, 16, or 32 histological slides are used for training. When limiting the number of training data in the Karolinska and Radboud datasets, only 4096, 8192, 16,384, 32,768, 65,536, 131,072, 262,144, or 524,288 randomly selected tile images, or all 972,800 and 782,336 images in the Karolinska and Radboud datasets, are used for training. In both training data limitation experiments, the last two encoder blocks are fine-tuned.

For augmentations and transformations, strong-augment (0.1.0) and albumentations (1.3.0)^59^ Python packages were used.

### Prostate cancer-specific death survival models

We evaluated PCa-specific survival using the 32 patient-level histology cluster fractions derived from the frozen prostate-m cluster dictionary. Three ridge-penalized Cox proportional hazards models were compared on identical patient splits: histology cluster fractions only, Gleason grade only, and histology cluster fractions plus Gleason grade. Gleason grade was represented as a categorical variable. Cluster fractions were standardized using the corresponding training data only.

Model development and evaluation used patient-level repeated nested cross-validation. The outer validation comprised 10 repeats of five stratified folds. Within each outer training set, 4-fold inner cross-validation performed all supervised operations, including model-specific ranking of the 32 cluster fractions, selection of the number of clusters, and selection of the ridge penalty. The outer test patients were not used for feature ranking, tuning, scaling, or model fitting.

Harrell concordance was the primary discrimination measure. The 15-year inverse-probability-of-censoring-weighted Uno concordance was evaluated as a secondary measure. We report mean outer-fold concordance, paired differences between models, and 95% confidence intervals corrected for dependence among repeated cross-validation folds.

## Results

### HistoEncoder—A foundation model for prostate cancer

HistoEncoder is a large neural network model consisting of a XCiT (Methods^17^, ^28^) pre-trained with the SSL procedure DINO.^22^ The training procedure leverages self-distillation to learn features, which are able to discriminate between images in the training dataset. Importantly, this procedure does not aim to create a model for a specific task, such as cancer prediction or tissue image segmentation. Features learnt by DINO during pre-training have instead been shown to be useful in a wide variety of tasks, either after fine-tuning with small amounts of domain-specific data, or without any fine-tuning. HistoEncoder allows for domain-specific pre-training even with small datasets and leverages histology-embedded features in unsupervised clustering of gigapixel WSIs for binary classification (cancer vs benign), multiclass subtyping (e.g., Gleason grading) or multimodal end-to-end framework (survival analysis). Fig. 1 shows an architectural overview of HistoEncoder. The model is freely available as a Python package including an easy-to-use standalone program at https://github.com/jopo666/HistoEncoder.

We pre-trained HistoEncoder models (“prostate-s” and “prostate-m”; Methods) with prostate tissue slide images from 11,226 tissue slides and 1307 PCa patients, constituting a dataset of 48 million tile images (HelsinkiProstate dataset; Table 1). In addition, we pre-trained a HistoEncoder model (“natural-s”) on natural images (ImageNet) for models' assessment and comparison.

To understand whether representations learnt by the models captured clinically relevant information, we extracted features from both models for 3.2 million prostate tissue images not used in pre-training (Radboud dataset; Methods) as well as compared them with the four common multiorgan-based FMs. Features from the HistoEncoder model pre-trained with tissue images, but without any information on cancer presence or grade, distinguished cancerous from benign tissue. All FMs trained with tissue images including HistoEncoder produced histological features that displayed separation between benign and cancerous tissues, and further stratified tile images by Gleason grades (Fig. 2a). In contrast, the model pre-trained with natural images displayed much less separation between tissue classes (Fig. 2a).

**Fig. 2:**
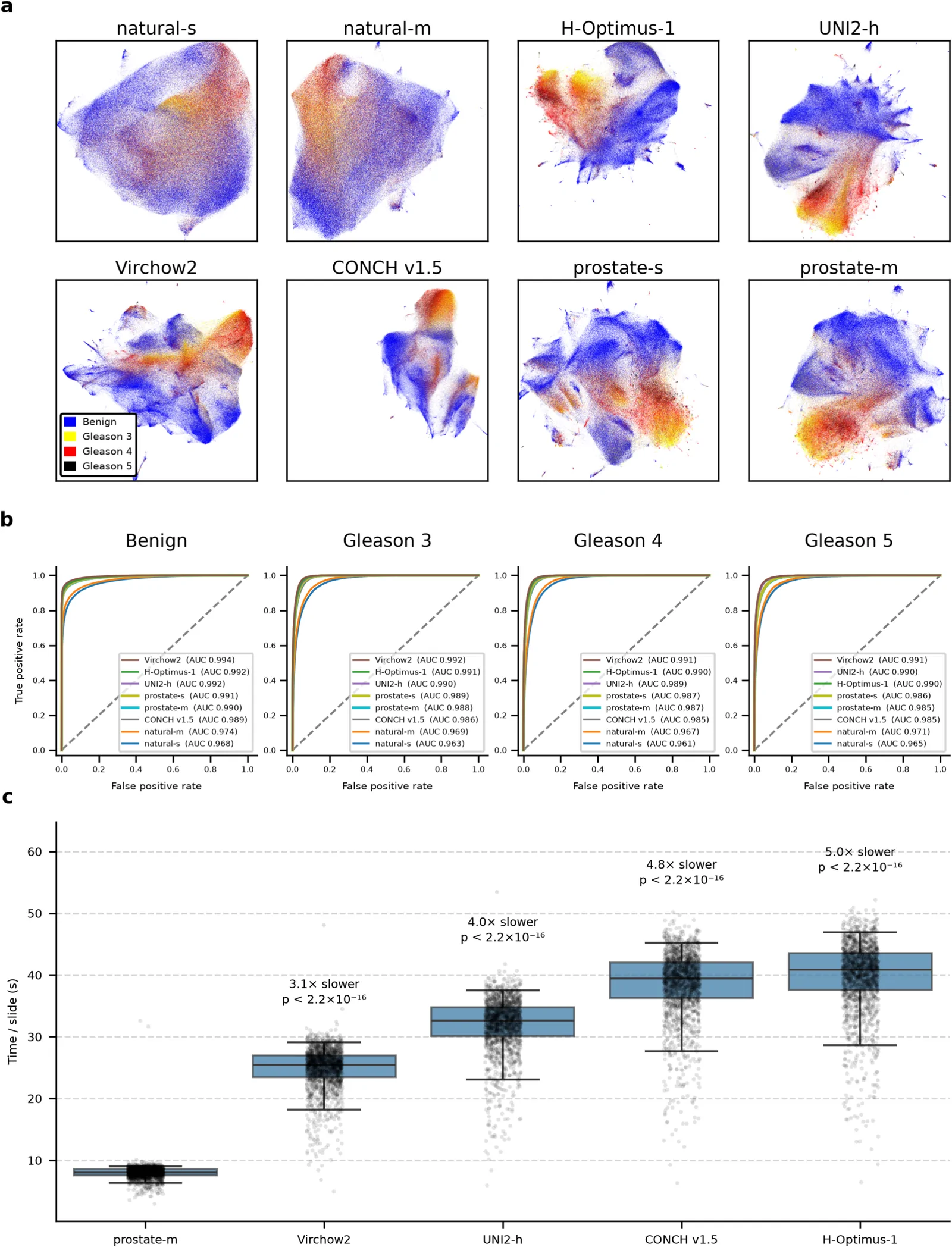
Benchmarking of HistoEncoder (prostate-s, prostate-m), foundation models trained with natural images (natural-s, natural-m), and four recent pan-tissue FMs (H-Optimus-1, UNI2-h, Virchow2, and CONCH v1.5). (a) UMAP representations of the features extracted from the epithelium tissue in the Radboud dataset. Benign and cancerous epithelium, as well as different Gleason grades, form clear clusters in the features extracted with the HistoEncoder (prostate-s, prostate-m) and pan-tissue models, whereas features extracted with the natural-s model are more mixed. (b) Receiver operating characteristic (ROC) curves for the tasks of classifying benign tissue and Gleason grades 3, 4, and 5 in the Radboud dataset with logistic regression on tile embeddings. Inset legend shows the area under ROC curve values in descending order for each method benchmarked. (c) Time taken by each model to extract features in the HelsinkiTMA dataset. Each point corresponds to a slide image. For each pan-tissue FM, a multiplier (e.g., 5.0×) shows the difference in processing time means compared to HistoEncoder/prostate-m. *p-*values shown for paired *t*-tests comparing per-slide image processing times.

We then assigned Radboud tile images into tissue classes with logistic regression classifiers built on features extracted with FMs. Both prostate-s and prostate-m achieved similar performance than the four pan-tissue FMs (Fig. 2b; Suppl. Table 5), whereas the natural image models performed slightly worse than the tissue-image trained models. Finally, we compared the feature extraction (inference) times of HistoEncoder prostate-m model with the pan-tissue models. As prostate-m is substantially smaller than the pan-tissue models, it was from 3× (Virchow2) to 5× (H-Optimus-1) faster on the average than the other models processing tile images (Fig. 2c, Supp. Table 4) while displaying similar performance in prostate tissue classification.

We then clustered tile images of two datasets not used in pre-training based on features extracted from the prostate-m model and annotated the clusters as malignant or benign (Methods). These clusters showed high label purity, with 54% of all cancerous tile images belonging to clusters where at least 90% of images were assigned either a malignant or benign label (Fig. 3a). The clusters also distinguished between stroma, epithelium, and malignant tissue (Fig. 3b). These findings highlight the ability of HistoEncoder to yield clinically informative features despite not being pre-trained on any clinical annotations or labels, and without any fine-tuning to adapt the method to a particular task, such as tissue classification.

**Fig. 3:**
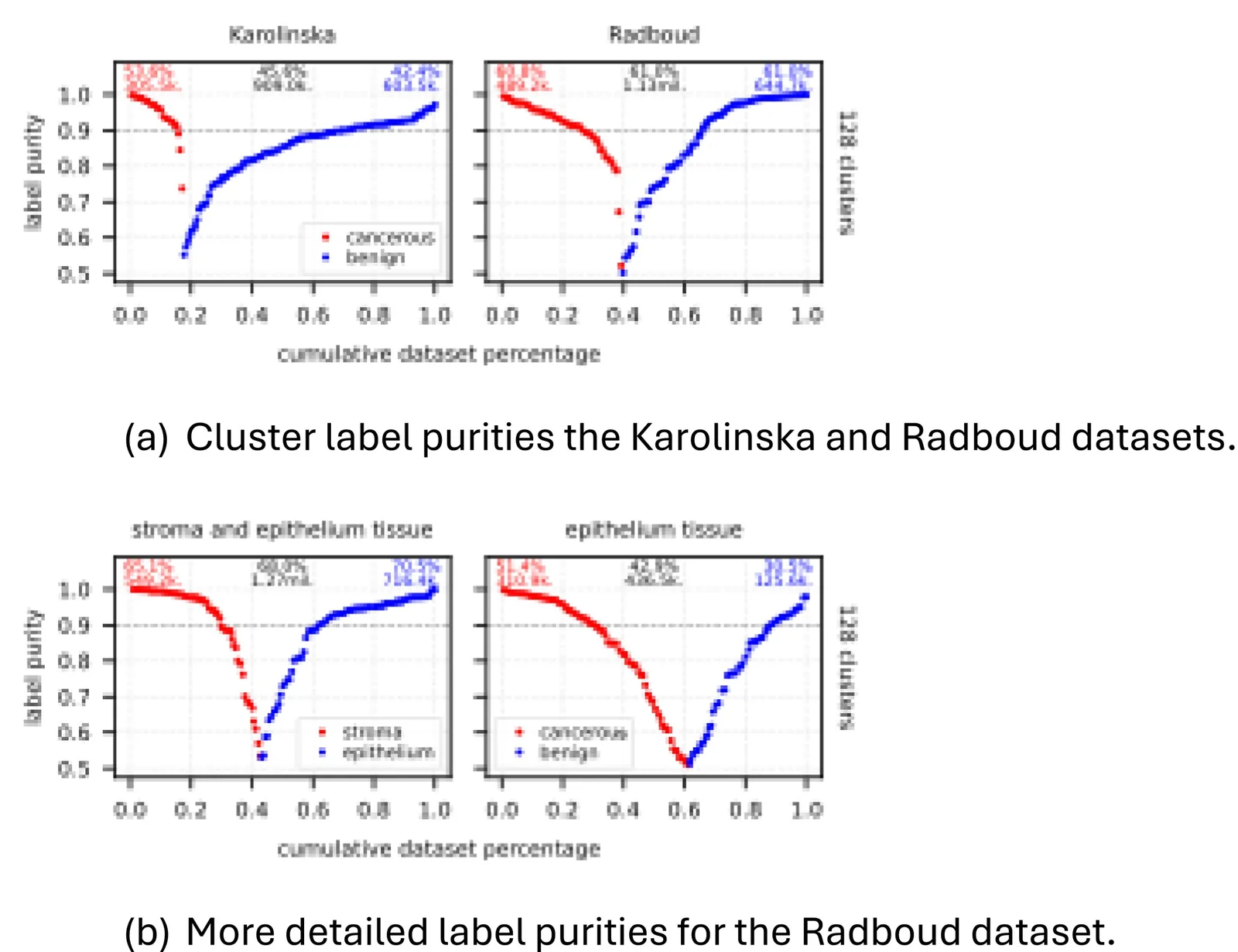
Cluster label purities for different labels in the Karolinska and Radboud datasets. A significant proportion of tile images in these datasets are contained in clusters with greater than 90% label purity for cancerous vs benign tissue (a), stroma vs epithelium tissue (b left), and cancerous vs benign epithelium tissue (b right).

### HistoEncoder outperforms natural image specific pre-training in predicting prostate cancer in tissue images even without fine-tuning

We next evaluated the performance of fine-tuned HistoEncoder models in predicting PCa presence on tissue slide images. HistoEncoder models pre-trained with tissue images were fine-tuned on datasets not used during training (Methods; “prostate-s” model). To understand the importance of domain-specific pre-training, we compared HistoEncoder models with encoder models (“natural-s”) that were identical to, and fine-tuned similarly as HistoEncoder models, but pre-trained on natural images.

HistoEncoder models fine-tuned with tissue images achieved 81%–95% AUROC tile-level classification performance, depending on the dataset used in fine-tuning (Fig. 4a; Suppl. Tables 1, 2, and 3). In contrast, the models pre-trained with natural images performed substantially worse (70%–92% AUROC). These models did not reach HistoEncoder performance even when only a small proportion of prostate-s model parameters were fine-tuned opposed to fine-tuning any number of natural-s model parameters (Fig. 4b; Suppl. Fig. 1 a). Interestingly, fine-tuning HistoEncoder models with only 4 slide images resulted in performance comparable to natural-s models fine-tuned with 37 slide images (Fig. 4b).

**Fig. 4:**
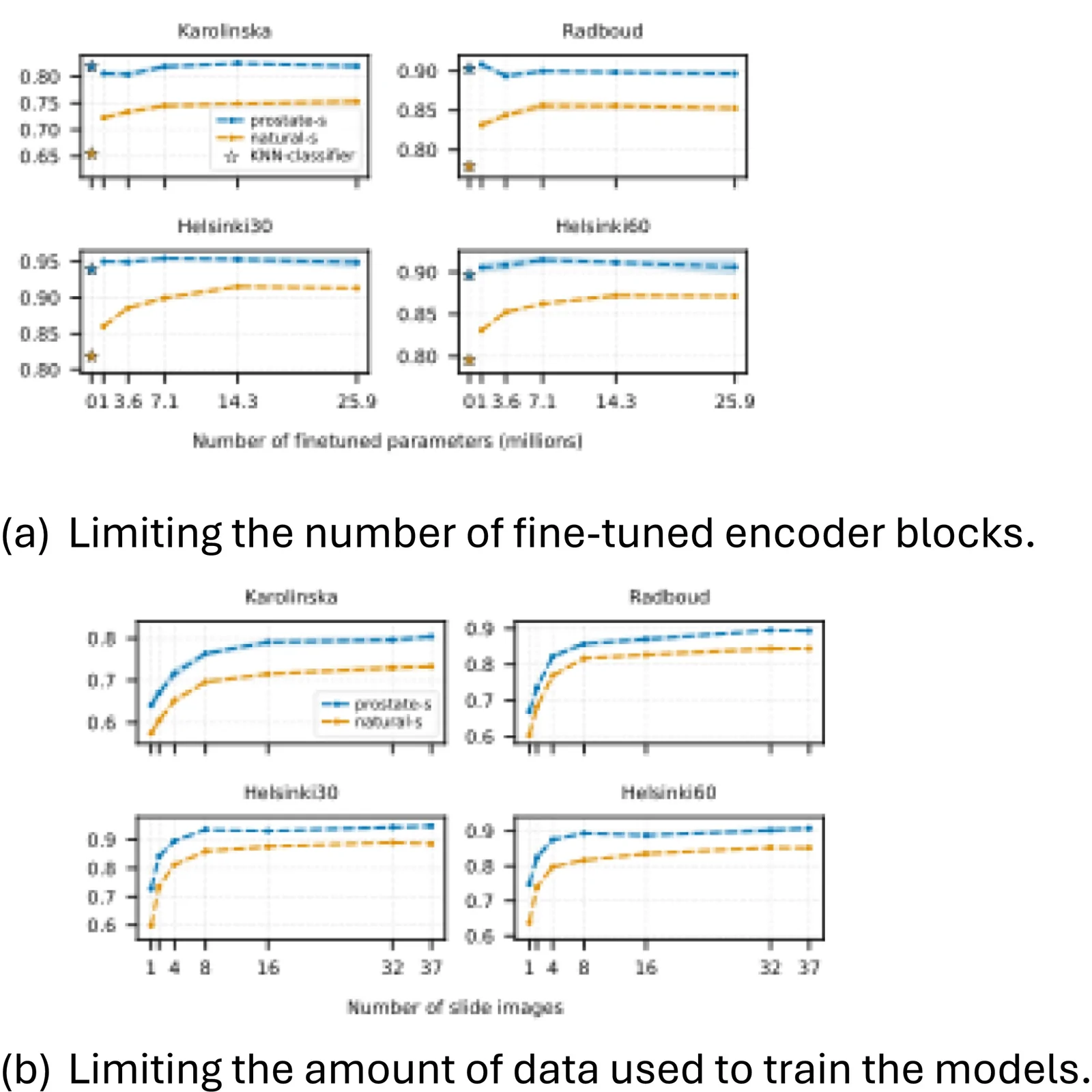
AUROC scores for the prostate cancer classifiers fine-tuned from pre-trained prostate-s and natural-s encoder models using the PESO dataset and evaluated against Karolinska, Radboud, Helsinki30, and Helsinki60 datasets. Prostate-s-based models achieve higher AUROC scores than natural-s-based models with less fine-tuned parameters (a) and training data (b). A simple KNN classifier requiring no training significantly outperforms a fully fine-tuned natural-s encoder model on all evaluation datasets. Note that prostate-s model has been pre-trained on Helsinki30 and Helsinki60 datasets, but not on Karolinska or Radboud datasets. Despite Helsinki30 and Helsinki60 being a part of the HistoEncoder training data, similar performance dynamics can be observed in all four evaluation datasets indicating that a FM pre-trained with tissue images is much more efficient to fine-tune with tissue images than an identical FM pre-trained with natural images.

We also evaluated the classification performance of prostate-s and natural-s models without any fine-tuning by constructing k-NN classifiers (Methods). Here, k-NN classifiers based on HistoEncoder features outperformed the corresponding natural-s k-NN classifiers, and remarkably, also all natural-s fine-tuned models (Suppl. Fig. 1 a). HistoEncoder models were found to benefit much more from fine-tuning data than models pre-trained with natural images, the latter requiring approximately 10 times more data before the performance tapered off (Suppl. Fig. 1 b).

### HistoEncoder achieves comparable prostate cancer-specific mortality prediction compared to Gleason grading

Accurate prediction of PCa-specific mortality is a clinically important yet markedly difficult problem. We thus set out to investigate whether HistoEncoder could predict PCa mortality and compare it to the current Gleason grades approaches in clinical use.

In repeated nested cross-validation, the histology-cluster-only model achieved a mean Harrell concordance of 0.695 (corrected 95% CI, 0.578 to 0.813), compared with 0.729 (corrected 95% CI, 0.651 to 0.807) for Gleason grade alone (Table 2). The paired difference for the cluster-only model relative to Gleason was −0.034 (corrected 95% CI, −0.153 to 0.086). Thus, the cluster fractions contained prognostic information of a similar magnitude to Gleason grading, but with substantial uncertainty in the estimates, and the analysis could not establish superiority.

**Table 2:** Internally cross-validated discrimination for prostate cancer-specific survival.

Model	Harrell C (95% CI)	Difference from Gleason (95% CI)	15-year Uno C (95% CI)	Difference from Gleason (95% CI)
HistoEncoder cluster fractions only	0.695 (0.578–0.813)	−0.034 (−0.153–0.086)	0.699 (0.561–0.836)	−0.031 (−0.160–0.098)
Gleason grade only	0.729 (0.651–0.807)	Reference	0.730 (0.645–0.815)	Reference
HistoEncoder cluster fractions plus Gleason grade	0.728 (0.651–0.806)	−0.001 (−0.026–0.024)	0.729 (0.647–0.812)	−0.001 (−0.026–0.025)

Values are means across 50 outer test folds from 10 repeats of 5-fold patient-level nested cross-validation. Model differences were paired on identical outer folds. Confidence intervals were corrected for dependence among repeated cross-validation folds. C, concordance; CI, confidence interval.

Adding cluster fractions to Gleason grade did not improve discrimination. The cluster-plus-Gleason model achieved a mean Harrell concordance of 0.728 (corrected 95% CI, 0.651 to 0.806), corresponding to a paired difference of −0.001 relative to Gleason alone (corrected 95% CI, −0.026 to 0.024). The 15-year Uno concordance analysis gave the same conclusion. The cluster-only model selected a median of four clusters (interquartile range, 4–6), whereas the cluster-plus-Gleason model selected zero clusters in 44 of 50 outer folds (88%). These results indicate that the cluster fractions capture survival-associated morphology, but provide no stable incremental predictive information once Gleason grade is available.

## Discussion

In this work, we introduced HistoEncoder, a FM for PCa DP. FMs hold significant promise to contribute to precision cancer medicine. Whereas pre-training a FM takes considerable computational resources, the model can then be fine-tuned to a multitude of specific tasks.^60^, ^61^, ^62^ Importantly, fine-tuning often requires orders of magnitude less data and resources than pre-training.^6^, ^63^, ^64^

We pre-trained HistoEncoder with 48 million tile images extracted from prostate tissue images and showed how the model could distinguish malignant and benign tissues, Gleason grades, as well as stromal and epithelial tissues without any annotated information on tissue types being available during training. In this study, we demonstrated how pre-training with domain-specific images yielded substantially better performance than using natural images for prostate histopathology tasks. In line with our findings, recent FMs trained on tissue images have had excellent performance in DP downstream tasks such as cancer detection and survival prediction. These models have, however, not challenged existing cancer-specific clinical prediction models to the extent that we address PCa.

Here, we demonstrated extending and applying HistoEncoder in two clinically relevant tasks. First, we created a classifier which achieved high performance in predicting the presence of PCa. We observed significantly better prediction accuracy, and compute and data efficiency with HistoEncoder models trained with tissue images compared to identical models trained with natural images, also in unsupervised cancer grading task. We also benchmarked HistoEncoder against four recent large pan-tissue H&E FMs, and observed that all performed at a consistently high level in Gleason grading. These results are compatible with the recent benchmarking effort by Akebli et al., who explored tissue image pathology FMs as a basis for PCa detection. It is important to note, however, that HistoEncoder models are much smaller in terms of model parameters and thus, much faster to execute than the leading pan-tissue models such as H-Optimus-1. This gives HistoEncoder models an advantage in resource efficiency and ability to perform a multitude of clinical analysis tasks in limited hardware settings. Second, we used HistoEncoder to summarize the distribution of histological patterns in each patient's tissue and evaluated these histological cluster fractions as predictors of PCa-specific mortality. In internal nested cross-validation, the image features alone achieved discrimination of a similar magnitude to conventional Gleason grading, showing that the self-supervisedly learnt representations capture prognosis-associated morphology. However, the confidence intervals were wide, the image-feature model did not outperform Gleason grading, and adding image features to Gleason grade did not improve discrimination. The prognostic information captured by the cluster fractions therefore appears to overlap substantially with that represented by Gleason grade in this cohort.

Previously, Pinckaers et al. developed a CNN model trained on BCR outcomes in a case–control setting.^65^ A biomarker derived from this ImageNet-pre-trained ResNet50-D model demonstrated significant predictivity in a multivariable model including pre-operative PSA, ISUP Gleason grade, pathological stage, and surgical margin status. In an external validation cohort, however, the conventional ISUP Gleason grade outperformed the biomarker. This highlights the challenge of developing generalizable models for real-world clinical applications.

A widely acknowledged challenge is heterogeneity in the histopathological ground truth across institutions and time periods, reflecting evolving grading recommendations, including known controversies between the 2019 ISUP and GUPS consensus statements for reporting PCa. Our datasets, as datasets in many previous publications, span different institutions and diagnostic eras, and the annotations or slide labels were not retrospectively rereviewed or harmonized. Annotation heterogeneity matters for grade-based downstream evaluation. However, it is less directly relevant to the self-supervised objective because Gleason, IDC-P, and cribriform labels are not used as pre-training targets. For these reasons, the main interest of the field and how the FM should be utilized is not to repeat Gleason grading with AI. Rather, finding new, potentially prognostic or predictive, features with the help of AI is the key objective in cancer diagnostics and treatment decision making. This reinforces the need for harmonized and well-curated cohorts that include long-term survival data.

We provide HistoEncoder as an easy-to-use Python package containing both the models pre-trained with prostate tissue images, as well as a standalone software tool to extract features and cluster tissue tile images. HistoEncoder can thus be extended to specific tasks involving any histopathological images.

As proof of concept, we were able to address clinical questions with HistoEncoder using limited data and computing resources from a single laptop computer. This exemplifies the utility of such FMs in lowering the barrier to creating clinical software tools. The prostate-s and prostate-m encoder models were able to compress information from large tissue areas into a feature vector, which was subsequently combined with other data modalities. If the other data modalities, such as spatial transcriptomics data^48^ or TMA spot labels, contain information for small tissue regions, feature vectors can be directly extracted for these regions with HistoEncoder models.

Taken together, our results demonstrate how HistofaEncoder can provide clinically relevant insights from tissue images. FMs such as HistoEncoder enable the rapid development of computational methods for precision cancer medicine, even with limited domain-specific data. Here, we showed that HistoEncoder performs at the same level as much more resource-intensive pan-cancer models in PCa detection, aligning with a previous study. An exciting direction would be to explore the utility of FMs in the analysis of multiple clinically relevant modalities and as part of multimodal predictor and interpreter models. Future multimodal models are likely to allow for leveraging tissue imaging on a large scale in conjunction with other clinical and molecular profiling data, leading to improved tools for cancer diagnosis, prognosis and treatment.

As it has been empirically demonstrated^21^, ^66^ that larger datasets and higher capacity SSL-pre-trained models will produce better performance, training on larger pathology datasets should be a priority. On the other hand, HistoEncoder provides clearly less resource-demanding FM for prostate-specific tasks such as domain-specific feature extraction, and performs on par with recently introduced pan-tissue FMs H-Optimus-1, Virchow2, UNI2, and CONCH that are trained with massive amounts of multiorgan pathology image data. HistoEncoder is thus a low-compute alternative to pan-tissue FMs, and easy to adopt to prostate-specific tasks. We envision combining HistoEncoder-derived features with electronic health record information to further increase its performance in clinically meaningful tasks.

## Author contributions statement

J.P.: Conceptualization, Methodology, Data curation, Formal analysis, Software, Writing – original draft. O.B.: Writing – review and editing, Formal analysis, Figure preparation. J.K: Writing – review and editing, Formal analysis, Figure preparation. A.R.: Project administration, Conceptualization, Resources, Supervision, Writing – review and editing, Funding acquisition. K.S. and A.E.: Data curation, Writing – review and editing. E.P.: Conceptualization, Writing – review and editing, Resources, Supervision. T.M.: Conceptualization, Writing – review and editing, Project administration, Resources, Supervision, Funding acquisition. All authors reviewed the manuscript.

## Code availability

HistoEncoder code, trained models (prostate-s, prostate-m) and easy-to-use Python package implementing the methods are available at https://github.com/jopo666/HistoEncoder.

## Declaration of competing interest

The authors declare that they have no known competing financial interests or personal relationships that could have appeared to influence the work reported in this article.

## Data Availability

Due to national legislation on secondary use of health data and tissue material, and institutional permissions for this research, individual-level clinical datasets cannot be made publicly available. Full de-identified clinical data and images underlying the findings of this study are available from the corresponding author upon request and subject to approval by the national authority (FIMEA) and Helsinki University Hospital. An earlier version of this manuscript has been posted as a preprint on *arXiv* (arXiv:2411.11458v2). Other publicly available datasets used in this study can be accessed through their respective publications. The original human subject and sample related data are available upon request, provided the institutional ethical approval and research permit allows data sharing according to the request. Restrictions may apply to the availability of the internal Helsinki University Hospital datasets, which cannot be made publicly available due to general data protection regulations, national legislation, and institutional guidelines. Publicly available datasets can be accessed through their respective publications. For data inquiries, please contact tuomas.mirtti@helsinki.fi.
