# Supplementary material for: HistoEncoder: A digital pathology foundation model for prostate cancer
Source: J Pathol Inform. 2026 Aug 31;23:100715. doi: 10.1016/j.jpi.2026.100715 (PMC13653481; doi:10.1016/j.jpi.2026.100715)
Supplement: Supplementary file 1 [file mmc1.pdf]

HistoEncoder: a digital pathology foundation model for prostate cancer

Supplementary Material

Supplementary Table 1. AUROC scores for a K-nearest neighbor prostate cancer classifier, fitted with features extracted from the PESO dataset.

Model	Parameters	Training method	Training data	Karolinska	Radboud	Helsinki30	Helsinki60
XCiT-S12	25.9	self-supervised	HelsinkiProstate	0.819	**0.903**	0.939	0.896
XCiT-M24	83.9	self-supervised	HelsinkiProstate	**0.824**	0.898	**0.943**	**0.901**
XCiT-S12	25.9	self-supervised	ImageNet	0.655	0.779	0.819	0.795
XCiT-M24	83.9	self-supervised	ImageNet	0.683	0.790	0.816	0.806
EfficientNet-B0	4.0	supervised	ImageNet	0.668	0.774	0.550	0.552
EfficientNet-B1	6.5	supervised	ImageNet	0.666	0.753	0.545	0.54
EfficientNet-B2	7.7	supervised	ImageNet	0.671	0.767	0.558	0.563
EfficientNet-B3	10.7	supervised	ImageNet	0.661	0.740	0.549	0.548
EfficientNet-B4	17.5	supervised	ImageNet	0.652	0.747	0.547	0.553
ResNet-18	11.2	supervised	ImageNet	0.682	0.762	0.547	0.553
ResNet-50	23.5	supervised	ImageNet	0.684	0.746	0.525	0.521
ResNet-101	42.5	supervised	ImageNet	0.618	0.676	0.531	0.531
XCiT-S12	25.9	supervised	ImageNet	0.645	0.690	0.707	0.714
XCiT-M24	83.9	supervised	ImageNet	0.647	0.720	0.752	0.739

Supplementary Table 2. AUROC scores for a K-nearest neighbor prostate cancer classifier, fitted with features extracted from the Karolinska dataset.

Model	Parameters	Training method	Training data	PESO	Radboud	Helsinki30	Helsinki60
XCiT-S12	25.9	self-supervised	HelsinkiProstate	0.936	0.916	**0.949**	**0.908**
XCiT-M24	83.9	self-supervised	HelsinkiProstate	**0.940**	**0.918**	0.945	0.902
XCiT-S12	25.9	self-supervised	ImageNet	0.857	0.811	0.821	0.808
XCiT-M24	83.9	self-supervised	ImageNet	0.837	0.790	0.796	0.790
EfficientNet-B0	4.0	supervised	ImageNet	0.685	0.778	0.526	0.544
EfficientNet-B1	6.5	supervised	ImageNet	0.707	0.778	0.529	0.551
EfficientNet-B2	7.7	supervised	ImageNet	0.692	0.770	0.523	0.544
EfficientNet-B3	10.7	supervised	ImageNet	0.744	0.784	0.541	0.551
EfficientNet-B4	17.5	supervised	ImageNet	0.704	0.749	0.541	0.547
ResNet-18	11.2	supervised	ImageNet	0.734	0.770	0.531	0.553
ResNet-50	23.5	supervised	ImageNet	0.710	0.718	0.529	0.530
ResNet-101	42.5	supervised	ImageNet	0.643	0.706	0.511	0.522
XCiT-S12	25.9	supervised	ImageNet	0.784	0.683	0.664	0.648
XCiT-M24	83.9	supervised	ImageNet	0.736	0.738	0.664	0.631

Supplementary Table 3. AUROC scores for a K-Nearest neighbor prostate cancer classifier, fitted with features extracted from the Radboud dataset.

Model	Parameters	Training method	Training data	PESO	Karolinska	Helsinki30	Helsinki60
XCiT-S12	25.9	self-supervised	HelsinkiProstate	**0.934**	**0.854**	**0.959**	**0.919**
XCiT-M24	83.9	self-supervised	HelsinkiProstate	**0.934**	0.852	0.956	0.910
XCiT-S12	25.9	self-supervised	ImageNet	0.875	0.757	0.831	0.818
XCiT-M24	83.9	self-supervised	ImageNet	0.865	0.756	0.819	0.804
EfficientNet-B0	4.0	supervised	ImageNet	0.768	0.740	0.545	0.545
EfficientNet-B1	6.5	supervised	ImageNet	0.764	0.736	0.538	0.537
EfficientNet-B2	7.7	supervised	ImageNet	0.745	0.721	0.537	0.537
EfficientNet-B3	10.7	supervised	ImageNet	0.773	0.718	0.559	0.550
EfficientNet-B4	17.5	supervised	ImageNet	0.676	0.653	0.499	0.501
ResNet-18	11.2	supervised	ImageNet	0.786	0.737	0.567	0.569
ResNet-50	23.5	supervised	ImageNet	0.780	0.711	0.551	0.543
ResNet-101	42.5	supervised	ImageNet	0.690	0.683	0.517	0.526
XCiT-S12	25.9	supervised	ImageNet	0.805	0.714	0.763	0.746
XCiT-M24	83.9	supervised	ImageNet	0.797	0.723	0.776	0.732

Supplementary Table 4. Runtime benchmarking of HistoEncoder and four recent pan-tissue foundation models (H-Optimus-1, UNI2-h, Virchow2, and CONCH v1.5) for feature extraction on the HelsinkiTMA Dataset.

**Encoder**	**Mean**	**Median**	**Std**	**Fold vs. Rep.**	**p-value**	**p-value fmt**
HistoEncoder	7.96	8.05	1.18	1.0	–	NaN
Virchow2	24.82	25.42	3.49	3.1	0	$< 2.2 \times 10^{-16}$
UNI2-h	31.87	32.63	4.60	4.0	0	$< 2.2 \times 10^{-16}$
CONCH v1.5	38.44	39.43	5.56	4.8	0	$< 2.2 \times 10^{-16}$
H-Optimus-1	39.79	40.87	5.86	5.0	0	$< 2.2 \times 10^{-16}$

Supplementary Table 5. Performance of logistic regression classifiers trained on tile embeddings for classifying benign tissue and Gleason grades 3, 4, and 5 in the Radboud dataset. Precision, recall, F1-score, and area under the receiver operating characteristic curve (AUC) are reported for each benchmarked method. Values are presented as point estimates with bootstrap confidence intervals.

**Model**	**Class**	**Precision**	**Recall**	**F1-score**	**AUC**
CONCH v1.5	Benign	0.9737 [0.9733–0.9742]	0.9868 [0.9865–0.9871]	0.9802 [0.9800–0.9805]	0.9892 [0.9890–0.9895]
	Gleason 3	0.7526 [0.7462–0.7588]	0.6329 [0.6263–0.6395]	0.6875 [0.6824–0.6928]	0.9863 [0.9859–0.9866]
	Gleason 4	0.7366 [0.7319–0.7412]	0.7360 [0.7313–0.7406]	0.7363 [0.7326–0.7400]	0.9847 [0.9844–0.9850]
	Gleason 5	0.7610 [0.7476–0.7745]	0.3936 [0.3824–0.4047]	0.5188 [0.5076–0.5300]	0.9845 [0.9838–0.9852]
	Macro	0.8060 [0.8020–0.8099]	0.6873 [0.6839–0.6908]	0.7307 [0.7273–0.7341]	0.9862 [0.9859–0.9864]
H-Optimus-1	Benign	0.9793 [0.9789–0.9797]	0.9878 [0.9875–0.9881]	0.9836 [0.9833–0.9838]	0.9924 [0.9923–0.9926]
	Gleason 3	0.7862 [0.7804–0.7920]	0.7014 [0.6952–0.7075]	0.7414 [0.7366–0.7461]	0.9906 [0.9904–0.9909]
	Gleason 4	0.7855 [0.7812–0.7898]	0.7860 [0.7817–0.7904]	0.7857 [0.7824–0.7891]	0.9898 [0.9895–0.9900]
	Gleason 5	0.7676 [0.7561–0.7790]	0.5337 [0.5224–0.5450]	0.6296 [0.6198–0.6394]	0.9903 [0.9897–0.9908]
	Macro	0.8296 [0.8263–0.8330]	0.7522 [0.7488–0.7555]	0.7851 [0.7820–0.7881]	0.9908 [0.9906–0.9910]
HistoEncoder-m	Benign	0.9761 [0.9757–0.9765]	0.9867 [0.9864–0.9870]	0.9813 [0.9811–0.9816]	0.9902 [0.9900–0.9904]
	Gleason 3	0.7701 [0.7639–0.7761]	0.6649 [0.6587–0.6714]	0.7137 [0.7087–0.7187]	0.9885 [0.9881–0.9888]
	Gleason 4	0.7492 [0.7447–0.7537]	0.7591 [0.7545–0.7636]	0.7541 [0.7507–0.7577]	0.9865 [0.9862–0.9868]
	Gleason 5	0.7444 [0.7312–0.7575]	0.4228 [0.4115–0.4342]	0.5393 [0.5282–0.5505]	0.9853 [0.9846–0.9861]
	Macro	0.8099 [0.8061–0.8138]	0.7084 [0.7050–0.7118]	0.7471 [0.7437–0.7504]	0.9876 [0.9874–0.9879]
HistoEncoder-s	Benign	0.9771 [0.9767–0.9775]	0.9874 [0.9871–0.9877]	0.9822 [0.9820–0.9825]	0.9909 [0.9907–0.9911]
	Gleason 3	0.7738 [0.7678–0.7797]	0.6803 [0.6741–0.6865]	0.7240 [0.7191–0.7289]	0.9889 [0.9886–0.9892]
	Gleason 4	0.7595 [0.7550–0.7640]	0.7648 [0.7603–0.7693]	0.7621 [0.7586–0.7657]	0.9872 [0.9869–0.9875]
	Gleason 5	0.7508 [0.7376–0.7637]	0.4307 [0.4195–0.4421]	0.5474 [0.5365–0.5584]	0.9859 [0.9852–0.9866]
	Macro	0.8153 [0.8115–0.8191]	0.7158 [0.7124–0.7192]	0.7539 [0.7506–0.7572]	0.9882 [0.9880–0.9885]
UNI2-h	Benign	0.9784 [0.9780–0.9788]	0.9875 [0.9872–0.9878]	0.9830 [0.9827–0.9832]	0.9919 [0.9917–0.9920]
	Gleason 3	0.7807 [0.7748–0.7865]	0.6928 [0.6868–0.6990]	0.7342 [0.7294–0.7389]	0.9899 [0.9896–0.9902]
	Gleason 4	0.7805 [0.7760–0.7850]	0.7764 [0.7721–0.7808]	0.7785 [0.7751–0.7819]	0.9890 [0.9888–0.9893]
	Gleason 5	0.7682 [0.7568–0.7797]	0.5327 [0.5213–0.5442]	0.6291 [0.6192–0.6391]	0.9904 [0.9899–0.9909]
	Macro	0.8270 [0.8235–0.8304]	0.7474 [0.7440–0.7508]	0.7812 [0.7781–0.7843]	0.9903 [0.9901–0.9905]
Virchow2	Benign	0.9827 [0.9823–0.9830]	0.9892 [0.9889–0.9895]	0.9859 [0.9857–0.9862]	0.9945 [0.9944–0.9946]
	Gleason 3	0.7984 [0.7929–0.8040]	0.7285 [0.7225–0.7344]	0.7618 [0.7573–0.7664]	0.9922 [0.9919–0.9924]
	Gleason 4	0.7937 [0.7895–0.7979]	0.8077 [0.8036–0.8118]	0.8006 [0.7973–0.8039]	0.9913 [0.9910–0.9915]
	Gleason 5	0.7704 [0.7587–0.7820]	0.5389 [0.5275–0.5505]	0.6342 [0.6241–0.6442]	0.9911 [0.9906–0.9916]
	Macro	0.8363 [0.8329–0.8397]	0.7661 [0.7627–0.7695]	0.7956 [0.7926–0.7987]	0.9923 [0.9921–0.9924]
natural-m	Benign	0.9585 [0.9580–0.9591]	0.9833 [0.9830–0.9837]	0.9708 [0.9704–0.9711]	0.9744 [0.9740–0.9749]
	Gleason 3	0.6918 [0.6841–0.6992]	0.4684 [0.4619–0.4751]	0.5586 [0.5525–0.5646]	0.9693 [0.9686–0.9701]
	Gleason 4	0.6377 [0.6325–0.6428]	0.6151 [0.6099–0.6201]	0.6262 [0.6220–0.6304]	0.9675 [0.9668–0.9681]
	Gleason 5	0.7042 [0.6842–0.7235]	0.1947 [0.1860–0.2038]	0.3051 [0.2933–0.3171]	0.9706 [0.9693–0.9719]
	Macro	0.7480 [0.7424–0.7533]	0.5654 [0.5623–0.5685]	0.6152 [0.6115–0.6188]	0.9705 [0.9700–0.9709]
natural-s	Benign	0.9530 [0.9525–0.9536]	0.9826 [0.9822–0.9829]	0.9676 [0.9672–0.9679]	0.9677 [0.9672–0.9682]
	Gleason 3	0.6685 [0.6607–0.6764]	0.4304 [0.4240–0.4370]	0.5237 [0.5174–0.5299]	0.9632 [0.9623–0.9641]
	Gleason 4	0.6257 [0.6202–0.6311]	0.5727 [0.5674–0.5779]	0.5980 [0.5936–0.6025]	0.9610 [0.9603–0.9617]
	Gleason 5	0.6827 [0.6612–0.7038]	0.1646 [0.1564–0.1729]	0.2652 [0.2535–0.2770]	0.9649 [0.9633–0.9665]
	Macro	0.7325 [0.7266–0.7384]	0.5376 [0.5346–0.5406]	0.5886 [0.5850–0.5923]	0.9642 [0.9636–0.9648]

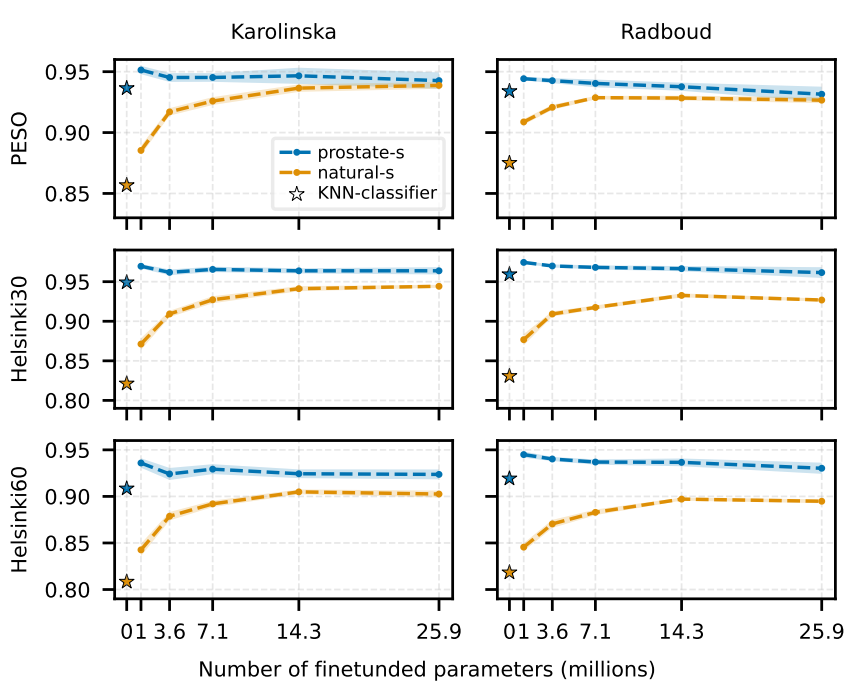

(a) Limiting the number of fine-tuned encoder blocks.

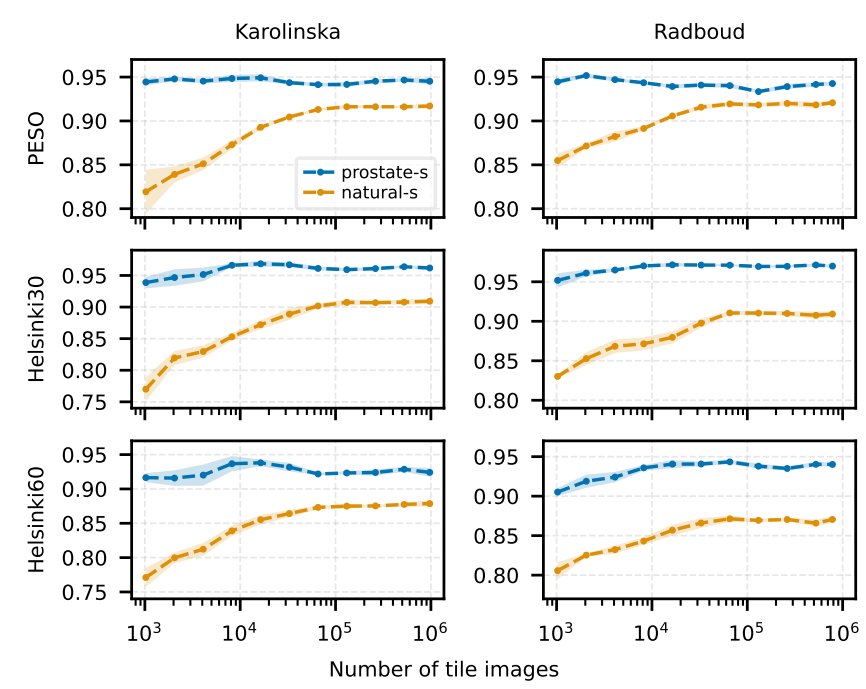

(b) Limiting the amount of training data.

Supplementary Figure 1. AUROC scores for the prostate cancer classifiers fine-tuned from `prostate-s` and `natural-s` encoder models using the Karolinska and Radboud datasets. `Prostate-s` based models achieve higher AUROC scores than `natural-s` based models with less fine-tuned parameters (*a*) and training data (*b*). A KNN-classifier achieves similar or significantly better performance than fully fine-tuned `natural-s` based models. When limiting the number of tile images (*b*), `prostate-s` based models significantly outperform `natural-s` based models even trained with 1000 times less data.
